# Dynamics of Polymorphism in a Malaria Vaccine Antigen at a Vaccine-Testing Site in Mali

**DOI:** 10.1371/journal.pmed.0040093

**Published:** 2007-03-13

**Authors:** Shannon L Takala, Drissa Coulibaly, Mahamadou A Thera, Alassane Dicko, David L Smith, Ando B Guindo, Abdoulaye K Kone, Karim Traore, Amed Ouattara, Abdoulaye A Djimde, Paul S Sehdev, Kirsten E Lyke, Dapa A Diallo, Ogobara K Doumbo, Christopher V Plowe

**Affiliations:** 1 Center for Vaccine Development, University of Maryland School of Medicine, Baltimore, Maryland, United States of America; 2 Malaria Research and Training Center, University of Bamako, Bamako, Mali; 3 Fogarty International Center, National Institutes of Health, Bethesda, Maryland, United States of America; World Health Organization, Switzerland

## Abstract

**Background:**

Malaria vaccines based on the 19-kDa region of merozoite surface protein 1 (MSP-1_19_) derived from the 3D7 strain of Plasmodium falciparum are being tested in clinical trials in Africa. Knowledge of the distribution and natural dynamics of vaccine antigen polymorphisms in populations in which malaria vaccines will be tested will guide vaccine design and permit distinction between natural fluctuations in genetic diversity and vaccine-induced selection.

**Methods and Findings:**

Using pyrosequencing, six single-nucleotide polymorphisms in the nucleotide sequence encoding MSP-1_19_ were genotyped from 1,363 malaria infections experienced by 100 children who participated in a prospective cohort study in Mali from 1999 to 2001. The frequencies of 14 MSP-1_19_ haplotypes were compared over the course of the malaria transmission season for all three years, in three age groups, and in consecutive infections within individuals. While the frequency of individual MSP-1_19_ haplotypes fluctuated, haplotypes corresponding to FVO and FUP strains of P. falciparum (MSP-1_19_ haplotypes QKSNGL and EKSNGL, respectively) were most prevalent during three consecutive years and in all age groups with overall prevalences of 46% (95% confidence interval [CI] 44%–49%) and 36% (95% CI 34%–39%), respectively. The 3D7 haplotype had a lower overall prevalence of 16% (95% CI 14%–18%). Multiplicity of infection based on MSP-1_19_ was higher at the beginning of the transmission season and in the oldest individuals (aged ≥11 y). Three MSP-1_19_ haplotypes had a reduced frequency in symptomatic infections compared to asymptomatic infections. Analyses of the dynamics of MSP-1_19_ polymorphisms in consecutive infections implicate three polymorphisms (at positions 1691, 1700, and 1701) as being particularly important in determining allele specificity of anti-MSP-1_19_ immunity.

**Conclusions:**

Parasites with MSP-1_19_ haplotypes different from that of the leading vaccine strain were consistently the most prevalent at a vaccine trial site. If immunity elicited by an MSP-1-based vaccine is allele-specific, a vaccine based on either the FVO or FUP strain might have better initial efficacy at this site. This study, to our knowledge the largest of its kind to date, provides molecular information needed to interpret population responses to MSP-1-based vaccines and suggests that certain MSP-1_19_ polymorphisms may be relevant to cross-protective immunity.

## Abstract 2

Christopher Plowe and colleagues surveyed local malaria parasites for genetic diversity in MSP-1, a candidate vaccine antigen. These data are needed to interpret population responses to MSP-1-based vaccines during trials planned at this site.

## Editors' Summary

**Background.:**

Malaria, a tropical parasitic disease, kills about one million people—mainly children—every year. Most of these deaths are caused by *Plasmodium falciparum,* which is transmitted to humans through the bites of infected mosquitoes. These insects inject a form of the parasite known as sporozoites into people that replicates inside liver cells without causing symptoms. Four to five days later, merozoites (another form of the parasite) are released from the liver cells and invade red blood cells. Here, they replicate 10-fold before bursting out and infecting other red blood cells. This massive increase in parasite burden causes malaria's flu-like symptoms. If untreated, it also causes anemia (a red blood cell deficit) and damages the brain and other organs where parasitized red blood cells sequester. Malaria can be treated with antimalarial drugs and partly prevented by reducing the chances of being bitten by an infected mosquito. In addition, researchers are developing vaccines designed to reduce the global burden of malaria. These contain individual malaria antigens (proteins from the parasite that stimulate an immune response) that should, when injected into people, prime the immune system so that it can rapidly control malaria infections.

**Why Was This Study Done?:**

The development of an effective malaria vaccine is not easy, in part because people can be simultaneously infected with several parasite strains. These often carry different variants (alleles) of the genes encoding antigens, which means that the actual parasite proteins might differ from the ones used for vaccination. If this is the case, the immune response generated by the vaccine might be less effective or even ineffective. An ideal vaccine would therefore stimulate an immune response that recognizes all these strain-specific antigens. However, little is known about their distribution in parasite populations in malarial regions, or about how this distribution changes over time (its dynamics). This information is needed to aid vaccine design and development. In this study, the researchers have investigated the distribution and dynamics of genetic variants of a merozoite antigen called MSP-1_19_, which is included in a vaccine currently being tested in Mali, West Africa. Although most of the MSP-1_19_ sequence is conserved, it contains six strain-specific polymorphisms (genetic variations); the candidate vaccine contains MSP-1_19_ from the 3D7 strain of P. falciparum.

**What Did the Researchers Do and Find?:**

The researchers used rapid DNA sequencing (pyrosequencing) to examine the MSP-1_19_ sequence in more than 1,300 malaria infections in 100 Malian children. They compared the frequencies of 14 MSP-1_19_ haplotypes (sets of polymorphisms at the six variant sites) over three years, in three age groups, and in consecutive infections within individuals. They found that the frequency of individual MSP-1_19_ haplotypes fluctuated in their study population but that those found in P. falciparum FVO and FUP strains were always the commonest, each being present in about 40% of the infections. By contrast, the P. falciparum 3D7 MSP-1_19_ haplotype was present in only 16% of the infections. They also found that mixed infections were more common at the start of each malaria season and in older individuals. In addition, individuals who were infected repeatedly by parasites from different strains (with different MSP-1_19_ variants) seemed to get sick with malaria more often than those infected multiple times by the same strain. The differences might, therefore, be important in determining the specificity of the immune response to MSP-1_19_.

**What Do These Findings Mean?:**

These findings indicate that most parasites that cause malaria at the Malian test site for the malaria vaccine that contains 3D7-specific MSP-1_19_ have a different form of MSP-1_19_. Although early results from field trials suggest that the 3D7-derived vaccine provides some protection against the more common FVO and FUP strains, the immunity stimulated by the vaccine might be mainly allele specific. If this turns out to be the case, these results suggest that a FVO- or FUP-derived vaccine might be more effective in Mali than the 3D7-derived vaccine, though not necessarily elsewhere. More generally, these results show the importance of determining the genetics of pathogen populations before starting vaccine trials. Without this information, a vaccine's ability to prevent infections with specific parasite strains cannot be determined accurately and potentially useful vaccines might be abandoned if they are tested in inappropriate populations. Importantly, baseline information of this sort will also allow vaccine developers to detect any vaccine-induced changes in the pathogen population that might affect the long-term efficacy of their vaccines.

**Additional Information.:**

Please access these Web sites via the online version of this summary at http://dx.doi.org/10.1371/journal.pmed.0040093.

- A related *PLoS Medicine* Perspective by Colin Sutherland discusses variation in malaria antigens as a challenge in vaccine development
- The malaria program of the University of Maryland Center for Vaccine Development performs research on many aspects of malaria
- Information on malaria and the development of vaccines is available from the Malaria Vaccine Initiative
- The World Health Organization provides links to general information on malaria plus some specific information on malaria vaccine development
- MedlinePlus encyclopedia has entries on malaria and on vaccination
- US Centers for Disease Control and Prevention provides information for patients and professionals on malaria
- US National Institute of Allergy and Infectious Diseases has information on malaria, including research into vaccines

## Introduction

Malaria remains a major cause of disease and death worldwide, killing millions every year [1]. The most severe form of the disease is caused by *Plasmodium falciparum,* which is responsible for most malaria mortality. Reducing the malaria burden will require the coordinated use of several strategies, including combination antimalarial therapies, insecticide-treated nets and, potentially, malaria vaccines [2].

Progress toward a malaria vaccine has been slow, owing in part to extensive genetic diversity in candidate vaccine antigens. As with other genetically diverse pathogens such as influenza virus, *Streptococcus pneumoniae,* and HIV, vaccines based on polymorphic malaria proteins may not elicit responses against all variants of the target antigen circulating in the parasite population. Immunization with such a malaria vaccine could lead to an increased frequency of variants not targeted by the vaccine. This phenomenon has been observed in trials of pneumococcal vaccines in which carriage of nonvaccine serotypes increased in vaccinated individuals [3,4], and continues to be a concern as nonvaccine serotypes have expanded following licensure of the seven-valent conjugate vaccine, PCV7 [5,6]. Influenza surveillance data are reviewed twice a year to determine which virus strains should be included in the vaccines for the upcoming transmission season [7]. A similar strategy has been proposed for development of HIV vaccines, whereby vaccine targets would be based on the genetic makeup of the viruses circulating in the target population [8]. However, the extensive genetic diversity and rapid evolution of these viruses could lead to the generation of vaccine-resistant strains. Malaria vaccine developers may face similar challenges.

Several subunit malaria vaccines in clinical development are based on merozoite surface protein 1 (MSP-1), the major protein on the surface of the extracellular blood stage of the parasite. The protein is synthesized as a 190 kDa precursor, which undergoes proteolytic cleavage into four fragments that remain on the surface of the merozoite as a glycosylphosphatidylinositol-anchored complex. Prior to erythrocyte invasion, the entire MSP-1 complex is shed, except for the C-terminal 19 kDa (MSP-1_19_), which remains on the merozoite's surface as it enters the erythrocyte [9]. MSP-1_19_ contains two epidermal growth factor (EGF)-like domains, which are thought to play a role in erythrocyte invasion [10]. Naturally acquired antibodies to MSP-1_19_ can inhibit erythrocyte invasion by preventing the secondary processing that releases this fragment from the rest of the MSP-1 complex [9,11,12], and are associated with protection from clinical malaria in field studies [13–18].

MSP-1_19_ has a highly conserved sequence, which along with its hypothesized critical function, makes it an attractive vaccine target. However, this region contains six nonsynonymous single nucleotide polymorphisms (SNPs): one in the first EGF-like domain corresponding to amino acid position 1644 (Q/E) and five in the second EGF-like domain corresponding to positions 1691 (K/T), 1699 (S/N), 1700 (N/S), 1701 (G/R), and 1716 (L/F) [19–23]. It is not clear what impact these polymorphisms have on anti-MSP-1_19_ immunity. Studies have demonstrated cross-reactive antibody responses between MSP-1_19_ haplotypes [24,25], but also some differential recognition [24–27], particularly of amino acids in the second EGF-like domain [24,25].

The FMP1/AS02A malaria vaccine, which is currently being tested in the field [28–30], is based on the C-terminal 42 kDa of MSP-1 from the 3D7 strain of P. falciparum [31] and contains amino acids ETSSRL at the six polymorphic residues in MSP-1_19_. Other P. falciparum strains are being targeted for MSP-1-based vaccines as well, including FVO and FUP, which have MSP-1_19_ haplotypes QKSNGL and EKSNGL, respectively. If immunity conferred by a vaccine is allele specific, then vaccination could increase the frequency of alleles not targeted by the vaccine and compromise vaccine efficacy. In a recent Phase I trial in Mali, West Africa, sera from adults immunized with a malaria vaccine based on the 3D7 strain reacted to FVO and FUP as well as to 3D7 parasites [30]. Only a few malaria vaccine candidates have been tested in efficacy trials powered to detect vaccine-induced selective effects [32–35]. The results of a Phase II trial of a blood stage vaccine suggested possible selection for clinical infections with nonvaccine type parasites in vaccinated individuals [34].

In preparation for efficacy trials of vaccines against malaria and other genetically variable pathogens, it is important to understand the distribution and natural dynamics of vaccine antigen polymorphisms in endemic populations. Molecular epidemiological studies will guide vaccine design and provide information that is needed to measure and interpret population responses to vaccines, both during efficacy trials and after introduction of vaccines into the population, and may provide insight into the selective forces (e.g., immunity) acting on antigen genes. In the largest study, to our knowledge, of malaria genetic diversity to date, we have determined the prevalence and dynamics of MSP-1_19_ haplotypes, both at a population level and within individuals, in a cohort of children and young adults residing at a malaria vaccine-testing site in Mali who were followed for three years during a malaria incidence study.

## Methods

### Study Site

Bandiagara is a rural town with 13,634 inhabitants in the Dogon Country of northeastern Mali. Mean annual rainfall is approximately 600 mm, and Anopheles gambiae is the primary vector for malaria. P. falciparum has intense, seasonal transmission corresponding to the July–October rainy season, with peak monthly entomological inoculation rates (EIRs) of up to 40–60 infected bites per person per month in September and total annual EIRs of 50–150. EIRs at the start and end of the transmission season (in June and December, respectively) are less than 1 infectious bite per person per month [36]. P. falciparum accounts for 97% of malaria infections, P. malariae for 3%*,* and P. ovale for rare infections*.* The disease burden is high, with children aged 10 y or less experiencing a mean of two clinical episodes of uncomplicated malaria a year [36] and a 2.3% annual incidence of severe malaria among children aged 6 y or less [37].

### Study Design

The samples used in this study were collected during a cohort study designed to measure the age-specific incidence of malaria infection and disease in children and young adults in Bandiagara. The study was conducted prospectively during the years 1999, 2000, and 2001. Study participants were aged 3 mo to 20 y and were recruited from all eight sectors of Bandiagara town in proportion to the populations of those sectors. From July to January of each year, individuals were followed actively by weekly visits. Each visit included a brief clinical examination, and those with symptoms consistent with malaria were given a full medical and laboratory assessment. Each participant contributed approximately 0.1 ml of blood, collected on 3MM Whatman filter paper (Whatman, http://www.whatman.com), at least monthly and at every episode of clinical malaria. Samples were collected under protocols reviewed and approved by Institutional Review Boards of the University of Maryland School of Medicine and the University of Bamako Faculty of Medicine. Informed consent was obtained from all study participants or their guardians.

For this study, 100 individuals with at least two years of follow-up during the malaria incidence study were randomly selected within three age strata. Thirty children aged 5 y or younger, 32 aged 6–10 y, and 38 aged 11 y or older were selected. Individuals selected for analysis did not differ from those not selected with respect to age, gender, or incidence of clinical malaria (unpublished data). Blood samples (*n* = 2,309) corresponding to all monthly surveys (*n* = 1,801) and clinical episodes (*n* = 508) occurring during the transmission season of the three years of the incidence study underwent DNA extraction (QIAamp DNA Mini Kit, Qiagen, http://www.qiagen.com) and PCR, and those yielding PCR products underwent pyrosequencing and haplotype estimation.

### PCR

As described previously [38], PCR to amplify the nucleotide sequence encoding MSP-1_19_ was performed on samples collected at all monthly surveys (regardless of microscopy results) and clinical episodes. A single PCR was used to amplify the region of interest from samples with parasitemia over 1,000 parasites/μl using biotinylated PCR primers (forward, 5′-CAATGCGTAAAAAAACAATGTCC-3′; reverse, 5′BIOTIN-TTAGAGGAACTGCAGAAAATACCA-3′). For samples with parasitemia below 1,000 parasites/μl and microscopy-negative samples, a nested PCR was used to amplify this region*,* using the above primers for the internal reaction and the following PCR primers for the external reaction: forward, 5′-TTAAACATTTCACAACACCAATG-3′; reverse, 5′-GAGTATTAATAAGAATGATATTCCTAAG-3′. Experiments comparing allele frequencies determined via single versus nested PCR showed no significant difference in allele frequencies (*n* = 64, mean difference in allele frequencies −0.5%, −0.2%, 1.6%, −0.7%, 0.4%, and −0.3% for the six SNPs of interest). The same reagent concentrations were used for both the single and nested PCR reactions and are described in a previous study [38], as are the cycling conditions for the single PCR. Cycling conditions for the external PCR included ten touchdown cycles beginning at a 63 °C annealing temperature with incremental changes of −0.5 °C/cycle, followed by 35 cycles with a 58 °C annealing temperature. The internal PCR used 2 μl of external PCR as template and ran for 25 cycles at a 60 °C annealing temperature. The 272 bp PCR products were visualized on 1.5% agarose gels. Samples that did not yield a product using the nested PCR were considered parasite-negative.

### Pyrosequencing

Using previously published methods, all PCR-positive samples underwent pyrosequencing to determine allele frequencies at each of the six SNPs in the nucleotide sequence encoding MSP-1_19_ [38]. Pyrosequencing (Biotage, http://www.pyrosequencing.com) is a high-throughput, real-time sequencing method that allows sequencing of short stretches of nucleotides (10–20 bp) surrounding known polymorphisms and quantification of the proportions of alternative nucleotides at each SNP. Four pyrosequencing reactions were used to genotype the six SNPs of interest.

### Haplotype Estimation

A mathematical model was used to estimate the frequency of 14 haplotypes in each genotyped sample given the allele frequencies generated by pyrosequencing [38]. The haplotype-estimating algorithm uses maximum likelihood methods to determine the most probable combination of haplotypes given the allele frequencies for an infection, the haplotypes known to be circulating in the population, and a probability distribution of the measurement errors.

### Statistical Analysis

Each parasite-positive sample (infection) was classified as having or not having each of the haplotypes. A haplotype was classified as being “present” if its frequency in the infection was above 10%. Infections could have more than one haplotype, and therefore, the sum of the prevalences of all the haplotypes was greater than one. Multiplicity of infection (MOI) was a count of the number of haplotypes in a given infection.

To investigate population-level dynamics, the prevalence of each MSP-1_19_ haplotype was compared over the course of the transmission season within each year, between the three years, and between the three age groups using chi-squared tests. Multivariable regression analysis was performed using generalized estimating equations (GEE) to model the log odds of each outcome of interest (MSP-1_19_ haplotype, MOI >1, and symptoms) as a function of time, age, and other covariates, and taking into account repeated measurements from the same individual.

To investigate within-host dynamics, each infection was classified as predominant or mixed with respect to the 14 haplotypes and with respect to each of the six polymorphic residues. A haplotype or individual amino acid was considered “predominant” in the infection if its frequency was over 60%, and infections without a predominant haplotype/amino acid were considered “mixed.”

Cox proportional hazards was used to model the time to next clinical episode in individuals' consecutive clinical infections as a function of change in predominant haplotype (or predominant amino acid at each polymorphic site), year, and age. The fixed effects partial likelihood method was used to determine the effect of having multiple events from the same individual.

Logistic regression was used to model the log odds of a change in predominant haplotype/amino acid in intervals consisting of an asymptomatic infection followed by a clinical episode versus intervals containing consecutive asymptomatic infections, adjusting for year, age, and interval length. GEE was also used in the same models to account for multiple intervals occurring in the same individual. No corrections were made for testing of multiple sites. For all analyses, only intervals occurring within a malaria transmission season were included; intervals spanning a dry season were not included. Statistical analysis was conducted using SAS (SAS Institute, http://www.sas.com).

## Results

### Prevalence and Population Dynamics of 14 MSP-1_19_ Haplotypes

**Figure 1:**
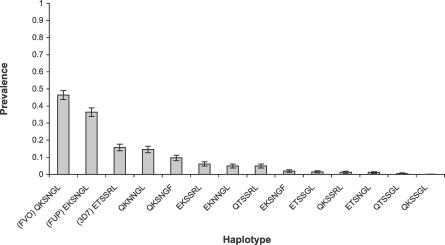
Prevalences of 14 MSP-1_19_ Haplotypes in a Cohort from Bandiagara, Mali Infections could have multiple haplotypes, thus haplotype prevalences do not sum to one. Haplotypes corresponding to the FVO, FUP, and 3D7 strains are indicated. Bars indicate 95% CIs.

Of the 2,309 samples collected from the study participants during the three transmission seasons, 1,375 were parasite-positive (by PCR). Of the samples positive for parasites by microscopy, 96% were PCR positive, and of microscopy-negative samples, 40% were PCR positive. Of the 1,375 parasite-positive samples, 1,369 gave successful genotyping results. A haplotype-estimating algorithm was used to determine the presence of 14 haplotypes in samples that yielded allele frequency data. The algorithm was able to resolve haplotype frequencies for 1,363 of the 1,369 genotyped samples. The overall prevalence of each haplotype in the 1,363 samples is shown in Figure 1. The QKSNGL and EKSNGL haplotypes (corresponding to the FVO and FUP strains, respectively) had the highest prevalence in the cohort with prevalences of 46% (95% confidence interval [CI] 44%–49%) and 36% (95% CI 34%–39%), respectively. The ETSSRL haplotype (corresponding to the 3D7 strain) had a lower overall prevalence of 16% (95% CI 14%–18%). Ten of the 14 haplotypes had prevalences below 10%, and eight haplotypes had prevalences below 5%.

**Figure 2:**
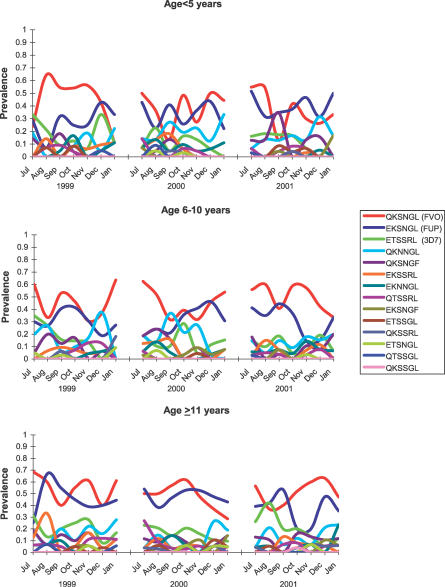
Prevalence of MSP-1_19_ Haplotypes over Time in Three Age Groups Haplotype prevalences from July to January by age group during the three years of the malaria incidence study in Bandiagara, Mali.

Figure 2 shows the prevalence of MSP-1_19_ haplotypes over time in three age groups. As seen in the figure, the frequency of individual haplotypes varied considerably over time. However, despite this variability, the QKSNGL (FVO) and EKSNGL (FUP) haplotypes were most prevalent during all three years and in all three age groups. In addition, it appeared that the difference in prevalence between the two most common types and the remaining types increased with age. The same patterns were observed when the analysis was limited to microscopy-positive samples (unpublished data).

### Multiplicity of Infection

**Figure 3:**
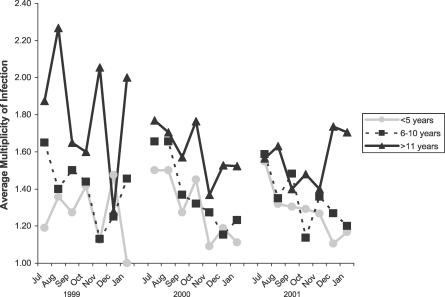
MOI by Age Average MOI over time in three age groups.

Figure 3 shows the average MOI in three age groups during the three years of the incidence study. Average MOI fluctuated over time, but overall tended to decrease over the course of the transmission season (Mantel-Haenszel Chi-square test for trend *p* = 0.074, *p* < 0.001, *p* = 0.027, for 1999, 2000, and 2001, respectively, for all age groups combined). In addition, MOI was higher in the oldest age group (≥11 y) than in the two younger age groups. In a multivariable regression model including month, study year, age, and parasite density and taking into account repeated measurements from the same individual, persons 11 years of age or older had a 2.7 times greater odds of having a mixed infection (defined as having more than one MSP-1_19_ haplotype) compared to children 5 years of age or younger (odds ratio [OR] 2.71, 95% CI 1.76–4.15, *p* < 0.001). The odds of having a mixed infection was not significantly different between the two younger age groups (OR 1.22, 95% CI 0.84–1.78, *p* = 0.24). The odds of a mixed infection was also greater in infections with moderate parasitemia (500–5,000 parasites/μl) compared to low-parasitemia infections (<500 parasites/μl) (OR 1.66, 95% CI 1.20–2.31, *p* = 0.0022). The odds of a mixed infection was not significantly different between higher-parasitemia infections and low-parasitemia infections (OR 1.11, 95% CI 0.80–1.55, *p* = 0.53 for 5,000–25,000 parasites/μl, and OR 1.07, 95% CI 0.75–1.53, *p* = 0.70 for >25,000 parasites/μl).

### MSP-1_19_ Haplotype Frequencies in Symptomatic and Asymptomatic Infections

**Figure 4:**
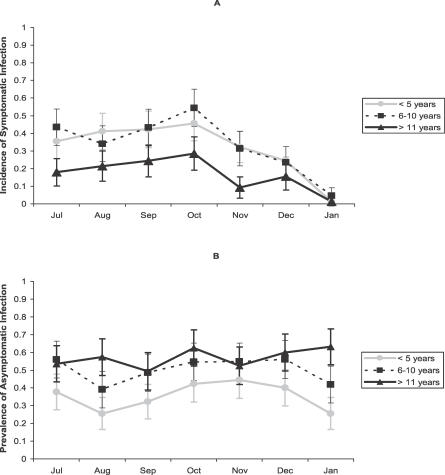
Frequency of Symptomatic and Asymptomatic Infections during the Malaria Transmission Season in Three Age Groups (A) Incidence of symptomatic infection over the course of the transmission season. (B) Prevalence of parasitemia among asymptomatic monthly surveys. Values in the figure represent the average for all three years of the study, since patterns were similar in all years. Bars indicate 95% CIs.

Figure 4 shows the frequency of symptomatic and asymptomatic infections in the cohort for all three study years combined. As seen in Figure 4A, the incidence of symptomatic infections peaked in October and decreased to almost zero by January, and was lower in individuals aged 11 years or older. Asymptomatic infection was less common in individuals aged 5 years or younger, and even in the absence of clinical malaria, asymptomatic parasitemia persisted, with a prevalence as high as 60% in the oldest individuals (Figure 4B).

**Figure 5:**
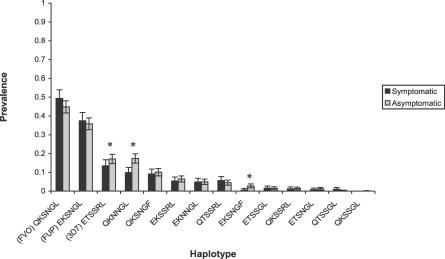
Prevalence of MSP-1_19_ Haplotypes among Symptomatic and Asymptomatic Infections Bars indicate 95% CIs. *Chi-square *p*-value = 0.08 (ETSSRL [3D7]), *p* < 0.001 (QKNNGL), and *p* = 0.006 (EKSNGF).

When the frequency of MSP-1_19_ haplotypes was compared between symptomatic and asymptomatic infections, three haplotypes (ETSSRL [3D7], QKNNGL, and EKSNGF) had a significantly lower prevalence among symptomatic infections compared to asymptomatic infections (Figure 5). The same results were observed in a multivariable model adjusting for time and age, while taking into account longitudinal measurements. With QKNSGL (FVO) as the reference group, the ETSSRL (3D7), QKNNGL, and EKSNGF haplotypes had decreased odds of being present in a symptomatic infection (ETSSRL [3D7]: OR 0.67, 95% CI 0.49–0.92, *p* = 0.012; QKNNGL: OR 0.48, 95% CI 0.35–0.68, *p* < 0.001; and EKSNGF: OR 0.29, 95% CI 0.09–1.02, *p* = 0.053). When parasite density was added to the model, these associations were no longer significant, suggesting that the associations between these three haplotypes and asymptomatic infection may be accounted for by differences in parasitemia. To test this hypothesis, GEE was used to model the association between each of the three haplotypes and parasite density, adjusting for time, age, and MOI. Indeed, both ETSSRL (3D7) and QKNNGL were more prevalent in the lowest-parasitemia infections (<500 parasites/μl) compared to the highest-parasitemia infections (>25,000 parasites/μl) (ETSSRL [3D7]: OR 2.12, 95% CI 1.41–3.20, *p* < 0.001; QKNNGL: OR 3.65, 95% CI 2.32–5.72, *p* < 0.001). QKNNGL was also more prevalent in moderate-parasitemia infections (500–5,000 parasites/μl) than in the highest-parasitemia infections (OR 2.44, 95% CI 1.38–4.31, *p* = 0.0021). There were no statistically significant associations between the EKSNGF haplotype and parasite density, although as one of the more rare haplotypes, the data may have been too few for meaningful multivariable analysis. The raw data indicate a frequency of 2.8% in the lowest parasitemia infections compared to a frequency of 0.9% in the highest frequency infections.

### Within-Host Dynamics of MSP-1_19_ Polymorphisms

**Figure 6:**
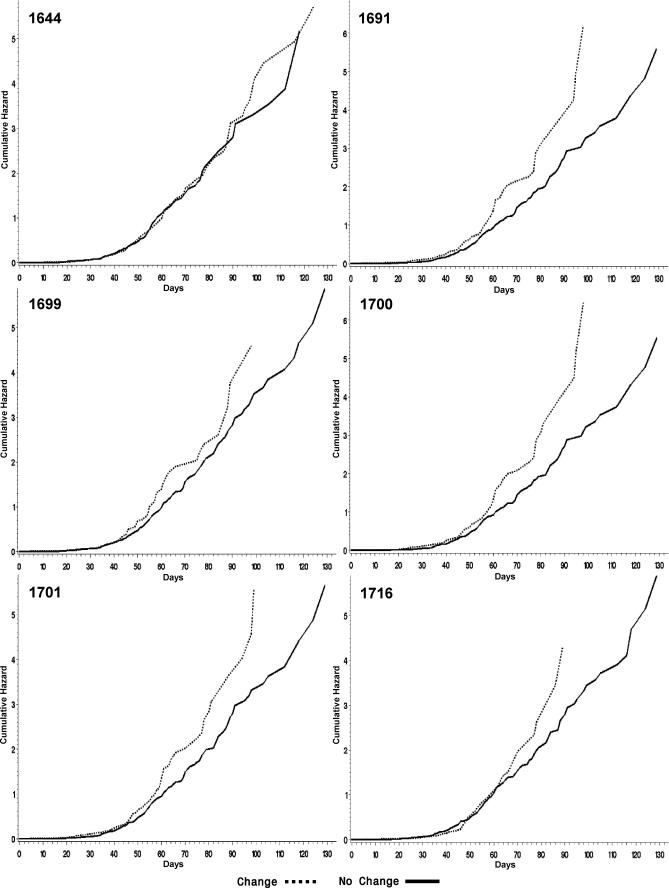
Cumulative Hazard of a Subsequent Clinical Infection according to Change in Predominant Amino Acid Cumulative hazard functions generated from Cox proportional hazards models of time to next clinical episode as a function of change in predominant amino acid at each polymorphic residue in MSP-1_19_, year, and age. The cumulative hazard is shown along the y-axis with days on the x-axis. Dashed lines indicate a change in predominant amino acid and solid lines indicate no change at positions 1644, 1691, 1699, 1700, 1701, and 1716. The hazard of a subsequent clinical episode was significantly greater when a change in predominant amino acid occurred at positions 1691, 1700 and 1701.

On average, individuals aged 5 years or less were parasite positive at 14 time points during the 3-y study period, seven of which corresponded to clinical episodes. Individuals aged 6–10 y were positive at 16 time points (six clinical episodes), and individuals 11 y and older were positive at 12 time points (three clinical episodes). Figure 6 shows cumulative hazard functions generated from Cox proportional hazards models of the time to next clinical episode in individuals' consecutive clinical infections as a function of change in predominant amino acid at each of the six polymorphic residues in MSP-1_19_, year, and age group. The hazard of a subsequent clinical infection when there was a change in overall haplotype was not significantly different from the hazard of a subsequent clinical infection when there was not a change in haplotype (hazard ratio [HR] 1.17, 95% CI 0.87–1.58, *p* = 0.30). However, as seen in graphs for amino acid positions 1691, 1700, and 1701 in Figure 6, when a change in predominant amino acid at each of the polymorphic residues was considered, the hazard of a subsequent clinical episode was greater when there was a change in predominant amino acid at these positions than when there was no change at these positions (1691: HR 1.48, 95% CI 1.13–1.94, *p* = 0.0044; 1700: HR 1.48, 95%CI: 1.13–1.93, *p* = 0.0039; 1701: HR 1.38, 95% CI 1.05–1.80, *p* = 0.021). The hazard of a subsequent clinical episode when there was a change at the other three polymorphic positions (1644, 1699, and 1701) was not significantly different from when there was no change (1644: HR 1.05, 95% CI 0.82–1.34, *p* = 0.72; 1699; HR 1.17, 95% CI 0.86–1.60, *p* = 0.32; 1716: HR 1.07, 95% CI 0.78–1.48, *p* = 0.67). These associations were not changed when mixed infections were excluded (i.e., going from a predominant to mixed infection was not considered a change) or when multiple events from the same individual were taken into account (unpublished data). In addition, the hazard of a subsequent clinical episode in 2000 and 2001 was less than the hazard in 1999 (2000: HR 0.53, 95% CI 0.38–0.74, *p* < 0.001; 2001: HR 0.56, 95% CI 0.41–1.76, *p* < 0.001), and as expected, was also less for individuals aged 11 y or more compared to children aged 5 y or less (HR 0.64, 95% CI 0.45–0.92, *p* = 0.016).

**Figure 7:**
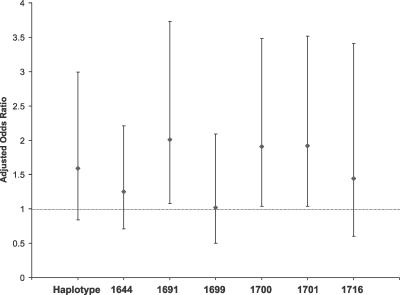
Association between Change in Predominant Haplotype or Amino Acid and Development of Clinical Symptoms ORs comparing the odds of a change in predominant haplotype or amino acid during intervals between two consecutive infections during which clinical symptoms developed, to the odds of a change during intervals between consecutive infections during which no symptoms developed, adjusting for year, age, and time between infections. Diamonds indicate point estimates and bars indicate 95% CIs. The dashed line designates an OR of 1, or no effect.

Figure 7 shows point estimates and confidence intervals comparing the odds of a change in predominant haplotype or predominant amino at each polymorphic residue in intervals including an asymptomatic infection followed by a symptomatic infection to intervals including consecutive asymptomatic infections, adjusting for year, age, and time between infections. The odds of a change in predominant amino acid at residues 1691, 1700, and 1701 were significantly greater in intervals ending with a clinical infection compared to intervals containing consecutive asymptomatic infections (1691: OR 2.01, 95% CI 1.08–3.73, *p* = 0.028; 1700: OR 1.91, 95% CI 1.04–3.48, *p* = 0.036; 1701: OR 1.92, 95% CI 1.04–3.52, *p* = 0.036). For changes of any type, the longer the amount of time between infections, the greater the odds of a change. The patterns were the same when intervals including mixed infections were excluded and when GEE was used to take into account multiple intervals from the same individual; point estimates were similar, but confidence intervals were wider, resulting in some associations no longer being significant at α = 0.05 (unpublished data).

## Discussion

To understand the natural variation in the frequency of polymorphisms in a malaria vaccine antigen, the nucleotide sequence encoding MSP-1_19_ was genotyped from 1,363 PCR-positive samples collected from 100 individuals who participated in a malaria incidence study at this site over three consecutive years. The two most prevalent haplotypes corresponded to strains of P. falciparum that are not included in a vaccine being tested in clinical trials at this and other sites in Africa, and although the frequency of individual MSP-1_19_ haplotypes fluctuated over time, these two haplotypes remained most prevalent during the three consecutive years of the study and in all three age groups. MOI was higher at the beginning of the transmission season and in older individuals. Three MSP-1_19_ haplotypes were associated with asymptomatic infection, which was accounted for by their being more prevalent in infections with low parasite density. Analyses of the dynamics of MSP-1_19_ polymorphisms within individuals' consecutive infections implicate three polymorphisms as being particularly important in determining allele specificity of anti-MSP-1_19_ immunity.

### Implications for Malaria Vaccine Design and Testing

By providing information about the prevalence and dynamics of vaccine antigen polymorphisms in a population being targeted for malaria vaccines, this study will inform choices about which MSP-1_19_ haplotypes to include in future vaccine formulations, and will allow more accurate interpretation of the efficacy of current formulations of MSP-1–based vaccines being tested in clinical trials. For example, the FMP1 vaccine antigen is based on the C-terminal 42 kDa of MSP-1 from the 3D7 strain of P. falciparum (MSP-1_19_ haplotype ETSSRL) [31]. In the Malian cohort examined in this study and in other malaria endemic areas, this haplotype is not the most common haplotype circulating in the parasite population [20,22,23]. The FVO (QKSNGL) and FUP (EKSNGL) haplotypes were the most prevalent in this study and the FUP (EKSNGL) haplotype had the highest frequency in Vietnam [20], Kenya [22], and Thailand [23]. If immunity conferred by such monovalent vaccines is allele specific, a vaccine with high allele-specific efficacy would have low overall efficacy in populations where the target allele is in the minority. Without an understanding of the distribution of vaccine target haplotypes, this scenario could result in the premature abandonment of a promising vaccine that could be modified (perhaps by including additional target alleles) to be more universally protective. This possibility highlights the need to include molecular endpoints in addition to conventional efficacy endpoints in clinical trials of malaria vaccines.

Analyses of the within-host dynamics of MSP-1_19_ polymorphisms indicate that changes in amino acids at positions 1691, 1700, and 1701 are associated with a shorter time to next clinical infection and with the development of clinical symptoms, suggesting that these residues may be particularly important in determining allele specificity of anti-MSP-1_19_ immune responses. These results are consistent with previous studies of anti-MSP-119 immunity, which suggest that there is some differential recognition of MSP-119 haplotypes [24–27], particularly based on polymorphic amino acids in the second EGF-like domain [24,25]. However, sera from malaria-experienced adults immunized with the 3D7-based FMP1/AS02A malaria vaccine reacted to FVO and FUP as well as to 3D7 parasites [30]. Coordinated molecular epidemiological and immunoepidemiological studies are needed to confirm the importance of these residues in determining the dynamics of MSP-1_19_ diversity, particularly at sites like ours where malaria transmission is seasonal, since MSP-1_19_ antibody responses wane quickly in the absence of exposure [39].

If the amino acids present at positions 1691, 1700, and 1701 do define an MSP-1_19_ “serotype,” then instead of 14 haplotypes, there would be six pertinent serotypes, with the KNG serotype (FVO and FUP) having a prevalence of 88% and the TSR serotype (3D7) having a prevalence of 20% in this Malian cohort. This finding greatly reduces the amount of genetic diversity vaccine developers would need to take into account in developing a polyvalent MSP-1–based vaccine, allowing them to focus on the residues most relevant to cross-protection.

### Natural and Vaccine-Induced Selection

While temporal variation in the prevalence of individual haplotypes was observed in the cohort from Mali, the relative frequency of the haplotypes remained stable—that is, the most prevalent haplotypes, FVO and FUP, remained most prevalent, and rare haplotypes remained rare. Our results contrast with the temporal changes observed in 138 isolates from Brazil, where in 1985–1986 the FUP haplotype was absent and the 3D7 haplotype was present in 40% of samples collected, but in 1997–1998 the FUP haplotype was more common than 3D7 (37% versus 10%, respectively) [19]. These different patterns may reflect true differences in haplotype dynamics in these locations, but may also have resulted from differences in sample size, sampling interval, and study duration.

It is possible that the stability of MSP-1_19_ polymorphism prevalences in Mali might be due to balancing selection acting to maintain genetic diversity at this locus, as has been demonstrated for other regions of MSP-1 [40–42]. If vaccine-induced immunity is allele specific, then vaccination could disturb this equilibrium, imposing directional selection that favors alleles not targeted by the vaccine. Such directional selection could induce changes in haplotype frequency and cause a reduction in vaccine efficacy over time, although this effect would likely be diluted by the substantial parasite population maintained in unvaccinated older children and adults. By decreasing the effective population size, vaccination could also increase the effect of genetic drift in the parasite population, resulting in decreased stability of haplotype frequencies. Use of insecticide-treated nets or prolonged effective drug treatment might be expected to have the same effect.

### Genetic Diversity and Malaria Epidemiology

Although MOI based on MSP-1_19_ is likely an underestimate of true MOI, the observed pattern of greater MOI in older individuals is in agreement with studies that have documented similar associations between MOI and age [43,44]. It has been hypothesized that fevers experienced during clinical malaria episodes may play a role in clearing parasitemia in younger children, while older children and adults have acquired immunity that limits disease but does not clear genotypes below the threshold of detection [43,44]. This hypothesis is consistent with the epidemiology of malaria in this area of Mali, where there is a lower incidence of symptomatic infection in individuals aged 11 years or more. However, the increased MOI in this group could also be due to less frequent treatment. Whatever the cause, these data emphasize the possibility that older children and adults serve as an important reservoir of genetic diversity.

MOI was highest at the beginning of the transmission season and decreased over the course of the transmission season, particularly in the two younger age groups. This initial decrease in MOI may be due to an increase in clinical malaria episodes that lead to drug treatment and clearance of parasites, followed by a continued decrease in MOI as EIR drops after the rains cease in October. Since vaccination could reduce MOI, as it has in previous malaria vaccine trials [35,45,46], it is important to document the reduced frequency of mixed infections over the course of the transmission season at this site to allow these natural fluctuations to be distinguished from the effects of a vaccine.

The associations between MOI, age, and time do not appear to be confounded by parasite density, though the data suggest that MOI is higher in infections with moderate parasitemias. The apparent decrease in MOI in higher-parasitemia infections may reflect the rapid growth of a disease-causing parasite clone overtaking the infection and masking the presence of minor alleles.

This study also demonstrated a reduced frequency of three specific MSP-1_19_ haplotypes in symptomatic infections compared to asymptomatic infections, including one haplotype (ETSSRL [3D7]) currently being targeted for vaccines [29]. This association appears to be due to these haplotypes having a higher prevalence in low parasitemia infections. Vaccination against haplotypes associated with favorable clinical outcomes instead of those associated with poor clinical outcomes could result in increased morbidity and mortality in the human population. Although analyses of the within-host dynamics of MSP-1_19_ diversity suggest that a change in amino acid at specific residues may be more important than having any particular haplotype, it will be important to examine the association between MSP-1_19_ haplotypes and clinical malaria in other endemic regions and in the context of genome-wide analyses to determine whether these three haplotypes are inherently less virulent (or are linked to important virulence factors) or whether these associations are an artifact of the haplotype dynamics at this particular site.

### Conclusions

This molecular epidemiology study provides information needed to accurately measure and interpret population responses to an MSP-1-based malaria vaccine in clinical trials of vaccine efficacy. It also provides insight into which MSP-1_19_ polymorphisms may be most relevant to cross-protective immunity and thus informs vaccine design. If immunity elicited by such a vaccine is allele specific, a vaccine derived from either the FVO or FUP strain might have better initial efficacy in settings where these MSP-1_19_ haplotypes predominate and haplotypes corresponding to the leading vaccine strain, 3D7, are less frequent. Given the polymorphic nature of vaccine antigens for P. falciparum and other genetically diverse pathogens, it is important to monitor pathogen populations before, during, and after introduction of vaccines to determine vaccine efficacy at the molecular level and to detect potential vaccine-induced changes in the pathogen population that could compromise vaccine efficacy.

## Supporting Information

### Accession Numbers

The sequence of *msp-1* from the 3D7 strain of P. falciparum was published with the P. falciparum genome sequence and can be found under GenBank (http://www.ncbi.nlm.nih.gov/) accession number XM_001352134.

## Abbreviations

CI: confidence interval
EGF: epidermal growth factor
EIR: entomological inoculation rate
GEE: generalized estimating equations
HR: hazard ratio
MOI: multiplicity of infection
MSP-1_19_: 19-kDa region of merozoite surface protein 1
OR: odds ratio
SNP: single nucleotide polymorphism
